# Mutational Analysis of Myoclonin1 Gene in Pakistani Juvenile Myoclonic Epilepsy Patients

**DOI:** 10.1155/2021/7509825

**Published:** 2021-04-20

**Authors:** Tayyaba Saleem, Arooj Mustafa, Nadeem Sheikh, Maryam Mukhtar, Mavra Irfan, Saira Kainat Suqaina

**Affiliations:** Cell and Molecular Biology Laboratory, Department of Zoology, University of the Punjab, Lahore 54590, Pakistan

## Abstract

Juvenile myoclonic epilepsy (JME) is the most prevalent and genetically heterogeneous form of epilepsy and accounts for 10–30% of all the cases worldwide. Ef-hand domain- (c-terminal-) containing protein 1 (*EFHC1*) encodes for a nonion channel protein and mutations in this gene have been extensively reported in different populations to play a causative role in JME. Linkage between JME and 6p11-12 locus has already been confirmed in Mexican and Dutch families. A case-control study was conducted on Pakistani JME patients for the first time, aimed at finding out *EFHC1* mutations that have been reported in different populations. For this purpose, 66 clinically diagnosed JME patients and 108 control subjects were included in the study. Blood samples were collected from all the participants, and DNA was isolated from the lymphocytes by the modified organic method. Total 3 exons of *EFHC1*, harboring extensively reported mutations, were selected for genotypic analysis. We identified three heterozygous variants, R159W, V460A, P436P, and one insertion in the current study. V460A, an uncommon variant identified herein, has recently been reported in public databases in an unphenotyped American individual. This missense variant was found in 3 Pakistani JME patients from 2 unrelated families. However, *in silico* analysis showed that V460A may possibly be a neutral variant. While the absence of a majority of previously reported mutations in our population suggests that most of the mutations of *EFHC1* are confined to particular ethnicities and are not evenly distributed across the world. However, to imply the causation, the whole gene and larger number of JME patients should be screened in this understudied population.

## 1. Introduction

Juvenile myoclonic epilepsy (JME) is the most prevalent form of genetic generalized epilepsy and accounts for at least 10–30% of all epilepsies [1, 2]. JME alone is responsible for 2 million cases in India [3]. No epidemiological data of JME is present for Pakistan, so the exact prevalence is not known yet. JME is typified by myoclonic jerks that predominantly occur after waking, generalized tonic–clonic (GTC) seizures, and infrequent absence seizures [4]. Epileptologists follow the varied criteria for JME diagnosis but the key diagnostic feature, according to all epileptologists is the same that all JME patients experience myoclonic seizures with or without GTCS and absence seizure in the early morning. Typical EEG symptom for JME is considered as polyspike wave pattern of >4 Hz with normal background activity. Seizures are well controlled when the patient follows the prescription on regular basis but upon discontinuation of medication, a high recurrence rate has been reported in various studies [5, 6].

JME seizures occur in adolescents with the typical onset age of 12-18 years [7]. It has been found more prevalent in females than in males (23). Till date, mutations in eight Mendelian genes (gamma-aminobutyric acid receptor subunit alpha-1; *GABRA1*, calcium-sensing receptor; *CASR*, gamma-aminobutyric acid receptor, delta; *GABRD*, calcium channel, voltage-dependent, beta-4 subunit; *CACNB4*, ef-hand domain- (c-terminal-) containing protein 2; *EFHC2*, bromodomain-containing protein 2; *BRD2*, sodium channel, voltage-gated, type i, beta subunit; *SCN1B*, and ef-hand domain (c-terminal)-containing protein 1; *EFHC1*) have been identified which are linked with JME [8, 9]. So far, heterozygous mutations in the coding sequence of *EFHC1*/*Myoclonin1* are persistent, causing JME including autosomal dominant, singleton, and sporadic cases in various independent families around the world [7, 10, 11]. Genetic studies have affirmed that mutations in *EFHC1* constitute 3 to 9% of all the JME cases all over the world [12]. *EFHC1* gene consisting of 11 exons encodes a 70 kDa protein of 640 amino acids containing three DM10 domains, a motif with unknown function, and an EF-hand Ca2+-binding motif. *EFHC1* modulates the apoptotic activity by interacting with R-type voltage-dependent calcium channels. Thus, the mutations in *EFHC1* interfere with the apoptotic activity of this gene by increasing the neuronal density leading to the production of hyperexcitable circuits [13].

Interestingly, EFHC1 mutations are not limited to JME but it has also been identified in different idiopathic generalized epilepsies and temporal lobe epilepsy. EFHC1 mutations may be considered pleiotropic due to their involvement in various epilepsy phenotypes [14]. EFHC1 (a microtubule-associated protein) plays a vital role in radial migration and cell division during cerebral corticogenesis. Functional analysis of various mutations has established that mutant EFHC1 impairs mitotic spindle organization and affects the morphology of radial glia that further leads to disruption of the radial and tangential migration. Mutations in the EFHC1 gene potentially produce structural brain anomalies due to disruption in brain development [15].

Several studies have indicated that mutations in neurotransmitter receptors and ion channel genes are linked with JME. The mutations that have been reported in these genes were private mutations, only found in single families and typically of de novo origin, that are not spotted in biological parents. Importantly, these de novo mutations (DNMs) were not detected in other family-based association studies for JME in the same or different ethnicities [16]. Consequently, mutations in genes coding for ion channel proteins cannot be accounted as the common cause of JME. *EFHC1* is considered as a potential candidate gene for JME and has been found very interesting in many aspects. First, *EFHC1* is the only gene in which mutations were found in many unrelated families with JME across the world, and second, no ion channel protein is coded by it as usually the case in epilepsy [17].

This presents a new perspective to the pathophysiology of JME and genetic epilepsy as a whole. However, Pinto et al. found 6p12–11 loci very heterogeneous and reported *EFHC1* mutations nonexistent in 112 Dutch patients of JME [18]. Hence, it can be presumed that JME may be caused by mutations in multiple genes, which may vary between populations of different ethnic origins. Mapping of 6p12–11 loci in a large Belize family with JME revealed a common *EFHC1* polymorphism present in higher frequency than in healthy individuals, cosegregating with juvenile myoclonic epilepsy. When the cell death analysis mediated by *EFHC1* was judged, it was evident that this common polymorphism had no effect on protein function, prospecting the involvement of other nearby mutations accountable for JME in this family [19]. Several studies had questioned the mutation-specific pathogenic effects of the *EFHC1* gene linked to JME. Subaran et al. highlighted that the pathogenicity of *EFHC1* mutations may depend on heterogeneity in terms of the genetic background of the ethnic group considered for the study. Furthermore, this study cautions us that compelling evidence is indispensable to ascribe causation [20]. Therefore, to ascertain whether the different variants of the *EFHC1* gene contribute to JME in Pakistani patients, we genotyped 3 important exons of this gene on which mutations had been reported extensively in previous genetic studies.

## 2. Materials and Methods

### 2.1. Subjects

66 Pakistani patients with JME were selected for the current study from two different hospitals of Punjab, Pakistan, who were visiting the outpatient department from September 2018 to November 2019. All JME patients were diagnosed by neurologists by the following criteria. The onset of myoclonic seizures varied between 8 and 20 years age bracket. Patients experienced short bilateral myoclonic jerks of shoulders and arms without losing consciousness just after awakening. Interictal electroencephalography (EEG) of the patients showed diffused, bilateral synchronous, and 4-6 Hz polyspike waves with normal background, provoked by photic stimulation [21, 22]. Furthermore, patients with focal seizures, mental retardation, or with a suggestion of any degenerative disease were excluded from this study. This study was approved by the Bioethics Committee of University of the Punjab, Lahore, Pakistan. Written informed consent was signed by all the patients/guardians before taking the blood sample. Moreover, 108 control subjects of the same ethnicity and geographic origin were included in the study. All the control subjects were healthy and had no history of any neurological disorder.

### 2.2. *EFHC1* Genotyping

Peripheral venous blood was collected from all the participants of this study in EDTA-coated tubes. DNA extraction was done by the modified organic method [12]. Each DNA sample was subjected to quantification and qualification before amplifying the PCR products. Total 3 exons carrying common SNPs rs3804505, rs3804506 (exon 3), rs137852777 (exon 4), and rs1266787 (exon 8) of *EFHC1* were targeted for mutational analysis in Pakistani JME patients. To amplify the PCR products, previously reported primers were used [23]. The purification of amplicons was done by using the purification kit of GeneJET PCR (Thermo Scientific™ #K0702) and sequenced commercially. All the sequence electropherograms of cases and controls were analyzed by BioEdit 7.2 software to find out variations. NCBI-BLAST was also performed to locate any change in the sequenced DNA samples. MEGA6 software was used to find the consequent amino acid changes. SNPs-GO and PhD-SNP were used for the *in silico* analysis of the identified variants.

## 3. Results

### 3.1. Clinical Characteristics

Our cohort of 66 JME patients contained 39 (59%) males and 27 (40%) females. The average age of onset for JME was observed 12 years varied between 10 and 19 years. Whereas in the control group, there were 64 males and 44 females. The mean age of the control group was found 15 years. Clinical characteristics of the JME patients are given in Table 1.

### 3.2. Mutation Analysis

**Figure 1:**
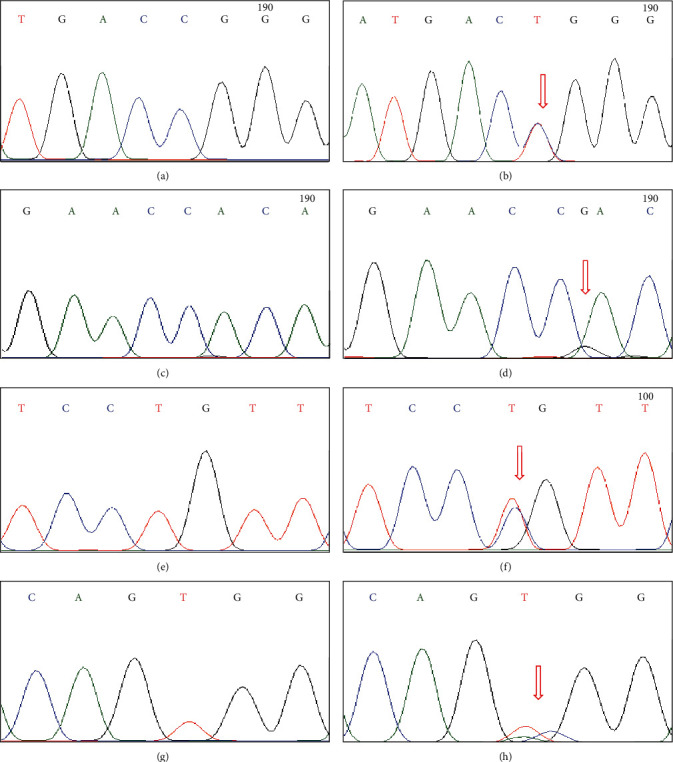
Electropherograms of identified variants. (a) Wild type and (b) R159W; (c) wild type and (d) c.723 + 18_723 + 19insG; (e) wild type and (f) P436P; (g) wild type and (h) V460A.

We identified 4 different variants in 66 JME patients, two of which were benign variants that had not been reported in dbSNP or any other database and are listed in Table 1. Two heterozygous variants were detected in exon 8; one in exon 3, while in noncoding region adjacent to exon 4 of *EFHC1*, 1 bp insertion was found. Missense heterozygous variant c.475C > T (rs3804506) found in exon 3 led to the change of arginine into tryptophan at 159 positions in the protein. It was observed in heterozygous form in JME patients and healthy control subjects. While a very rare heterozygous missense variant c.1436 T > C (rs764251038) and a benign synonymous variant c.1365 T > C were detected in 3 and 2 Pakistani JME patients, respectively. No other JME patients or control subjects carried these variants. However, we were not able to collect the blood samples of the whole family of these probands. Sequences of both of these novel variants were submitted to ClinVar-NCBI and assigned with accession number. Reference SNP (rs or RefSNP) number assigned by dbSNP to these variants is also given in Table 2. Electropherograms of all the variants detected in the current study are given in Figure 1 with wild type and mutant alleles.

## 4. Discussion

**Figure 2:**
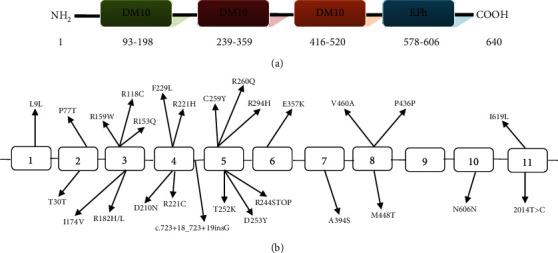
(a) Diagrammatic representation of *EFHC1* protein. (b) Genomic arrangement of *myoclonin1* gene. Previously reported mutations along with novel variants identified in Pakistani JME patients are shown in different exons. Novel variants detected in the Pakistani patients are given in bold type.

Our study showed slight male predominance in JME patients. Gender distribution is cogitated to be equal but some studies have manifested female preponderance [24]. Total 674 single nucleotide polymorphisms including 132 exonic variants have been identified in the myoclonin1 gene and can be accessed through the human genome database (GENOME 1000). Till date, many pathogenic mutations, including missense variant, deletions in the promoter region, nonsense variant, and frameshift variant, have been studied in different populations around the globe but not a single study from Pakistan added data to this database [10, 22]. This is the first study that investigated the variants of the *EFHC1* gene with reference to JME in the Pakistani population. JME prevalence has not yet been evaluated in Pakistan, but on the base of our experience of blood sampling for JME patients, we can presume that JME has a lower prevalence compared to other idiopathic epilepsies in this Asian region. We evaluated 66 Pakistani JME patients for the *EFHC1* variants and unearthed 4 variants in total 3 exons and their immediately adjacent intronic regions. The identified variants are presented in Figure 2 along with previously reported mutations. All these variants were considered as singleton as the mode of inheritance was not determined due to lack of blood samples from families. The result of this study are negative for R182H (exon 3), D210N, R221C, R221H, F229L (exon 4), and M448T (exon 8) that have been reportedly found in Hispanic, Indian, Dutch, African American, and Caucasian JME patients [13, 18, 23]. It can be conferred that *EFHC1* mutations are not evenly distributed in different ethnicities.

Missense variant R159W located on exon 3 was found both in JME patients and healthy controls, so it was considered as a polymorphism. Consistent with the findings of Ma et al., the frequency of this polymorphism did not differ in both subjects in the Pakistani population, so it cannot be considered as a major predisposing factor for JME [25]. In another study conducted on the Hispanic population, it was observed that R159W occurred almost at equal frequency in patients and control subjects [23]. Pinto et al. also observed this polymorphism in Dutch families but failed to find any statistically significant difference at allelic and genotypic level [18]. In the current study, 1 bp insertion was also found in the intronic region immediately adjacent to exon 4 in one JME patient of this study. This insertion was not reported before the current study in any ethnicity and possibly is benign in nature. A very rare heterozygous missense variant V460A was found in 3 JME patients belonging to two unrelated families. Two of these JME patients were siblings. This family had 3 JME affected individuals with unaffected parents, but the unavailability of blood samples from all the members limited our effort to study the inheritance pattern of this very rare variant. V460A is a coding missense variant recently been submitted to The Single Nucleotide Polymorphism Database (dbSNP) by The Exome Aggregation Consortium (ExAC) and The Genome Aggregation Database (gnomAD) Exomes in unphenotyped individuals. It was found neutral variant when pathogenicity of this variant was checked by SNPs-GO, PhD-SNP, and several other *in silico* tools. One novel synonymous variant (P436P) was also identified in the two JME patients of this study and was not found in any control subject.

To imply the causality, the altered function of protein caused by any of the novel variant should be monitored by biological assays. The same notion can be expanded to all the novel missense variants identified in any earlier studies, which are considered pathogenic on the basis of cosegregation and prevalence among JME patients.

## 5. Conclusion

Uneven distribution of *EFHC1* mutations among different ethnicities explains the importance of the genetic background of the patient for a mutation to play a causative role. We identified only one previously reported pathogenic variant in the current study, indicating that some other genes at the EJM1 locus might be involved in JME susceptibility in this population. In the present study, only 3 exons of the *EFHC1* gene were analyzed, other exons and the promoter region of this gene remained to be evaluated, which may harbor the disease-causing mutations. Assertion of *EFHC1* genetic contribution to JME necessitates the evaluation of complete gene at a larger scale coinciding with the inheritance pattern in Pakistani patients.

## Figures and Tables

**Table 1:** Clinical characters of the JME patients.

Character	*N*
Gender	66
Female	27
Male	39

Age years (mean ± SD)	17 ± 3.33
Female	16 ± 3.12
Male	15 ± 3.24

Seizure type	
Myoclonic seizures	66
GTCS	50
Absence seizures	6
Absence+myoclonic	2
Absence + myoclonic + GTCS	4
Myoclonic+GTCS	46
Myoclonic seizures alone	14

Distribution of myoclonic jerks	
Upper extremities	57
Lower extremities	09

Precipitating factors	
Sleep deprivation	39
Fatigue	21
Stress	11

Family history	
1^st^-degree relatives	11
2^nd^-degree relatives	17

**Table 2:** Variants identified by sequencing the exons of *EFHC1* in Pakistani JME patients.

No.	Region	NCBI dbSNP ID	Nucleotide change	Amino acid change	Molecular consequence	Genotype counts/(no. of subjects)	
						JME probands	Control subjects
1	Exon 3	rs3804506	475C > T	R159W	Missense	19/66	11/108
2	Intron 5	rs1581829739	c.723 + 18_723 + 19insG	N/A	Unknown	1/66	0/108
3	Exon 8	rs764251038	1436 T > C	V460A	Missense	3/66	0/108
4	Exon 8	rs1581846971	1365T > C	P436P	Synonymous	2/66	0/108

## Data Availability

The data used to support the findings of this study is available upon request from the corresponding author.
